# Prognostic and Predictive Value of CpG Island Methylator Phenotype in Patients with Locally Advanced Nonmetastatic Sporadic Colorectal Cancer

**DOI:** 10.1155/2014/436985

**Published:** 2014-01-14

**Authors:** Yuwei Wang, Yadong Long, Ye Xu, Zuqing Guan, Peng Lian, Junjie Peng, Sanjun Cai, Guoxiang Cai

**Affiliations:** ^1^Department of Colorectal Surgery, Fudan University, 270 Dong An Road, Shanghai 200032, China; ^2^Department of Oncology, Shanghai Medical College, Fudan University, 270 Dong An Road, Shanghai 200032, China

## Abstract

*Purpose*. In the present study, the prognostic significance of CpG island methylator phenotype (CIMP) in stage II/III sporadic colorectal cancer was evaluated using a five-gene panel. *Methods*. Fifty stage II/III colorectal cancer patients who received radical resection were included in this study. Promoter methylation of p14ARF, hMLH1, p16INK4a, MGMT, and MINT1 was determined by methylation specific polymerase chain reaction (MSP). CIMP positive was defined as hypermethylation of three or more of the five genes. Impact factors on disease-free survival (DFS) and overall survival (OS) were analyzed using Kaplan-Meier method (log-rank test) and adjusted Cox proportional hazards model. *Results*. Twenty-four percent (12/50) of patients were characterized as CIMP positive. Univariate analysis showed stage III (*P* = 0.049) and CIMP positive (*P* = 0.014) patients who had significantly inferior DFS. In Cox regression analysis, CIMP positive epigenotype was independently related with poor DFS with HR = 2.935 and 95% CI: 1.193–7.220 (*P* = 0.019). In patients with CIMP positive tumor, those receiving adjuvant chemotherapy had a poor DFS than those without adjuvant chemotherapy (*P* = 0.023). *Conclusions*. CIMP positive was significantly correlated with decreased DFS in stage II/III colorectal cancer. Patients with CIMP positive locally advanced sporadic colorectal cancers may not benefit from 5-fluorouracil based adjuvant chemotherapy.

## 1. Introduction

Colorectal cancer is a major cause of mortality and morbidity throughout the world. With adjuvant chemotherapy as standard management following surgery to treat stage III and stage II patients with high risk factors, the 5-year relative survival rate of locally advanced colorectal cancer was still 69.2% compared with 90.1% among patients with localized disease [1], which highlighted the need of better prognostic and predictive markers to identify those high-risk individuals.

Promoter CpG island hypermethylation resulting in the transcriptional silencing of tumor suppressor genes has been widely observed in colorectal cancer and been increasingly recognized to contribute to the pathogenesis of colorectal cancer. The subset of colorectal cancers with exceptionally high frequency of CpG island methylation were referred to as CpG island methylator phenotype (CIMP) [2] and showed distinct clinicopathological characteristics [3–5]. Tumor-specific hypermethylated loci of p14ARF, hMLH1, p16INK4a, MGMT, and MINT1 were proved to be closely related with colorectal cancers [2, 6]. However, the prognostic and predictive value of CIMP in sporadic locally advanced colorectal cancer determined by the five-gene panel had not been evaluated before.

Our study was designed to investigate the prognostic effect of CIMP epigenotype in stages II and III sporadic colorectal cancer interacting with adjuvant chemotherapy in Chinese population utilizing the five-gene panel. The aim of the study was to provide evidence contributing to risk stratification and individualized management for patients with locally advanced sporadic colorectal cancer.

## 2. Materials and Methods

### 2.1. Patient Selection

Between July 2004 and November 2004, 50 patients with stage II/III colorectal cancer who had received curative resection and were pathologically confirmed as adenocarcinoma or mucinous adenocarcinoma at the Colorectal Surgery Department, Fudan University Shanghai Cancer Center, were included for analysis. None of the patients were diagnosed as hereditary colorectal cancer (Lynch syndrome or familial adenomatous polyposis) or malignant tumors of other organs. Patients who underwent preoperative chemoradiation therapy or were treated with local excision were excluded. Eighteen and 32 patients were staged into stages II and III, respectively, according to the current TNM staging system of the 7th edition of the American Joint Committee on Cancer's (AJCC) Staging Manual [7]. Thirty-six patients completed six-month adjuvant chemotherapy using either standard mFOLFOX6 or CapeOX regimen. The clinicopathological features of the enrolled patients were summarized in Table 1. Informed consents were given to the patients prior to sample collection. This study was approved by the Ethics Committee of the Fudan University Shanghai Cancer Center.

### 2.2. Tissue Samples Collection and Genomic DNA Extraction

Fresh tissue samples were collected within half an hour after the removal of tumor from the patient and were stored at −80°C. Fresh frozen tissues of colorectal cancer were reviewed pathologically to ensure that the sample tissue contained more than 80% cancer cell proportion. Genomic DNA was extracted using EZNA Tissue Kit (Omega Bio-Tek, Norcross, GA, USA) according to manufacturer's instructions. A260 and A280 of the DNA samples were tested to measure the quantity and purity of genomic DNA.

### 2.3. Bisulfite Modification and Methylation Specific PCR

Bisulfite modification of 500 ng genomic DNA was performed using EZ DNA methylation kit (Zymo Research, Orange, CA, USA) according to the manufacturer's instructions. Methylation specific PCR (MSP) was performed as described previously [8]. Briefly, 50 ng of bisulfite-modified genomic DNA, standard reaction buffer, 10 pmol of each primers, and 0.75 units of Hotstart Taq DNA polymerase (Qiagen, GmbH) were mixed into a total volume of 25 *μ*L. Amplifications were carried out in a thermal cycler (Perkin-Elmer, Foster City, CA, USA). Primer sequences and cycling conditions for methylated and unmethylated strand of p14ARF, hMLH1, p16INK4a, MGMT, and MINT1 were described in previous study [8]. PCR products were separated by 2% agarose gel and visualized under UV illumination.

CIMP classification was based on the number of methylated genes of the panel markers. Tumors were classified as CIMP positive if 3 or more markers were methylated or CIMP negative if none or less than 3 methylated markers were observed.

### 2.4. Statistical Analysis

Tumor size and age were categorized into two groups using median as cut-off value. Tumors located from the cecum to transverse colon were classified into proximal colon cancer. Tumors located from the left colonic flexure to rectum were categorized into distal colorectal cancer. Correlations of CIMP epigenotype with categorical clinicopathological variables were assessed using Chi-square test or Fisher's exact probability tests as appropriate. Comparisons of continuous variables were performed using Mann-Whitney *U* test.

The primary clinical outcome was disease-free survival (DFS). DFS was defined as the time from surgical resection of colorectal cancer to the date of the local recurrence or first distal metastasis confirmed pathologically or by clinical imaging or to the last follow-up date. Overall survival (OS) was defined as the time from surgery to the date of patient's death or to the last follow-up date. The impact factors of DFS and OS were analyzed using Kaplan-Meier method (log-rank test) and adjusted Cox proportional hazards models, respectively. Hazard ratios (HRs) and 95% confidence intervals (CIs) for DFS and OS were estimated using Cox regression. Furthermore, stratified survival analysis was performed according to CIMP epigenotype.

All *P* values presented were two sided. A *P* value <0.05 was regarded as statistically significant. All statistical tests were performed using SPSS (version 20.0, SPSS Inc, Somers, NY, USA) software package.

## 3. Results

### 3.1. Clinicopathological and Molecular Features

CIMP positive epigenotype was detected in 24.0% (12/50) of patients. Representative results of the electrophoresis of MSP products can be seen in our previous paper [8]. The correlations between clinicopathological characteristics and CIMP epigenotype are summarized in Table 1. CIMP positive was found to be associated significantly with proximal site (*P* = 0.046), stage III disease (*P* = 0.022), and poorly differentiated tumor (*P* = 0.007). Age, sex, histology, lymphovascular invasion, perineural invasion, preoperative CEA level, and adjuvant chemotherapy were not significantly correlated with CIMP epigenotype.

### 3.2. Influence of CIMP Epigenotype on DFS

The median DFS was 60 months. Three local recurrences were identified. Nineteen cases of distal metastasis were confirmed before the last follow-up date. Liver metastasis was most common, occurring in 9 patients while the lung was the second common metastatic organ involving 6 patients. The 5-year disease-free survival was 58% in all patients.

**Figure 1:**
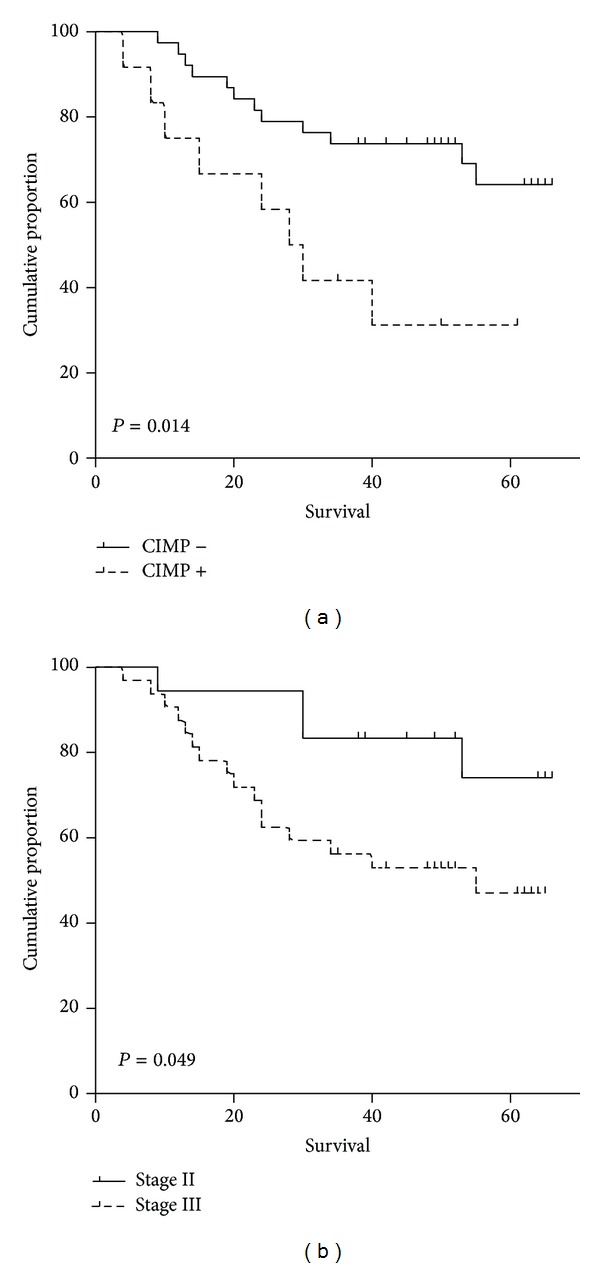
Disease-free survival analysis according to stage and CIMP status. Patients with CIMP positive tumors had a marginally significant poor DFS than those with CIMP negative tumors with *P* value =0.014 (a). Patients with stage II disease showed better DFS than those with stage III disease with *P* value =0.049 (b). DFS: disease-free survival, CIMP: CpG island methylator phenotype.

In the univariate analysis, TNM stage and CIMP epigenotype were the only variables which showed significant impact on DFS. Patients with stage III disease (5-year DFS: 47.1% versus 74.1%, *P* = 0.049, Table 2, Figure 1(a)) and CIMP positive epigenotype (5-year DFS: 31.3% versus 64.1%, *P* = 0.014, Table 2, Figure 1(b)) presented worse DFS. None of the analyzed variables had significant influence on OS (Table 3). In the multivariate analysis, CIMP epigenotype was the only independent prognostic factor on DFS (HR = 2.935, 95% CI: 1.193–7.220 and *P* = 0.019, Table 2).

### 3.3. Analysis of the Interaction between CIMP Epigenotype and Adjuvant Chemotherapy

**Figure 2:**
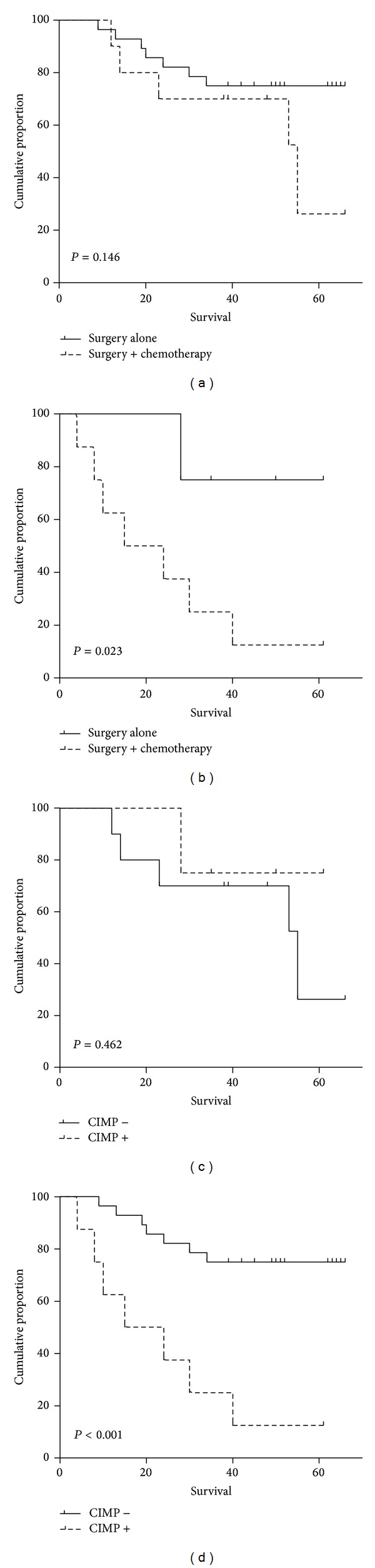
Disease-free survival analysis stratified by CIMP status and treatment jointly. In CIMP negative tumors, adjuvant chemotherapy showed no effect on DFS with *P* value =0.146 (a). Chemotherapy decreased DFS in CIMP+ tumors significantly with *P* value =0.023 (b). CIMP status showed no effect on DFS in patients treated with surgery alone with *P* value =0.462 (c). In patients receiving surgery plus adjuvant chemotherapy, CIMP positive patients had a significantly poor DFS than those negative with *P* value <0.001 (d). DFS: disease-free survival, CIMP: CpG island methylator phenotype.

Among CIMP negative patients, adjuvant chemotherapy had no effect on DFS (*P* = 0.146, Figure 2(a)). Surprisingly, among patients with CIMP positive tumors, those undergoing adjuvant chemotherapy had a decreased DFS significantly (*P* = 0.023, Figure 2(b)). In patients receiving surgery, CIMP epigenotype had no impact on DFS (*P* = 0.462, Figure 2(c)). However, in patients administrated with surgery plus adjuvant chemotherapy, CIMP positive epigenotype resulted in a significant reduction of DFS (*P* < 0.001, Figure 2(d)).

## 4. Discussion

In the present study, we identified a cohort of 50 stage II/III colorectal cancer patients treated with curative surgery alone or curative surgery followed by 5-FU based adjuvant chemotherapy and characterized the patients as CIMP positive or CIMP negative by performing methylation specific PCR (MSP) of 5 genes.

The prevalence of CIMP positive was reported to be 9%–90% in colorectal cancer and ranged widely between studies of populations of different ethnic backgrounds [9]. In our cohort, 24.0% of CIMP positive cases were detected. Clinicopathological features previously reported to be associated with CIMP positive epigenotype include female sex, older age of diagnosis, proximal colon, poor differentiation, mucinous carcinoma, low frequency of KRAS mutation, and high frequency of BRAF mutation [10, 11]. CIMP positive colorectal cancers enrolled in this study showed a predilection for proximal site and poor differentiation in consensus with previous reports [10, 11].

Furthermore, the prognostic effect of clinicopathological variables and CIMP status on stage II/III colorectal cancer was investigated. Our data suggested an unfavorable prognosis in patients with CIMP positive tumors and stage III tumors (Figure 1). In the Cox regression analysis, only CIMP epigenotype was significantly correlated with the DFS. In consistent with other studies [3, 12–16], our findings highlighted that the influence of CIMP status on DFS was independent of TNM stage and other clinicopathological variables included in the multivariate analysis (Table 3). In the study of Kim et al. [16], 320 cases of colorectal cancer were analyzed using MethyLight assay, and CIMP positive was proved to be significantly associated with poor prognosis. Inconsistent with most reports, Ogino et al. [17] suggested that patients with CIMP-high tumors experienced a significantly lower colon cancer-specific mortality adjusted for other prognostic factors.

In metastatic or recurrent colorectal cancer treated with 5-fluorouracil based chemotherapy, Ogino et al. [18] and Shen et al. [19] reported that CIMP positive defined a group of cases with markedly reduced overall survival. These results indicated a potential role of CIMP status in chemotherapy resistance of colorectal cancer. However, the interaction between CIMP epigenotype and adjuvant chemotherapy in stage II/III colorectal cancer remains controversial. Jover et al. [20] examined a population based cohort of 302 stage II/III CRC patients and found that those patients with positive CIMP did not benefit from 5-fluorouracil based adjuvant chemotherapy. The benefit from adjuvant chemotherapy was limited to patients with CIMP negative tumors. Evidence of our study also showed that in patients with CIMP positive stage II/III disease, patients treated with adjuvant chemotherapy showed inferior DFS than those without adjuvant chemotherapy. However, the results varied in other studies. Study of Van Rijnsoever et al. [21] suggested that CIMP positive predicted survival benefit from 5-fluorouracil based adjuvant chemotherapy independently in 103 stage III colorectal cancer patients. The evidence of Min et al. [22] indicated a positive effect of chemotherapy on DFS in CIMP positive stage II/III colorectal cancer. Heterogeneous results on prevalence, prognostic effect, and predictive value of CIMP epigenotype in sporadic colorectal cancer may be due to no consensus standard to define CIMP regarding panel gene markers, thresholds to define CIMP positive, and techniques to measure methylation status of maker genes [9]. In fact, there has been no consensus on the definition of CIMP positive yet. Different authors defined tumors that had methylation of two of three [21, 23], three of five [12, 24], three of six [25], or three of seven [2] genes as CIMP positive. The criterion adopted in our study referred to previous studies [2, 12, 26]. Actually, if the positive threshold to two or more of five genes reduced, the impact of CIMP on DFS lost its significance (*P* = 0.193).

Small sample size within each subgroup categorized by CIMP status, TNM stage, and chemotherapy limited the detection of the difference in prognostic significance. Potential selection bias could not be excluded in this retrospective study. It is noteworthy that the prognostic influence of CIMP status on stage II tumors and potential interactions with lymphovascular invasion, perineural invasion, and other clinical high risk factors of stage II colorectal cancer remains unclear since only 18 stage II cases were include in our study. The clinical decision-making regarding the adjuvant treatment of stage II is on the basis of identifying high-risk individuals by assessing the clinicopathological high risk factors. However, conventional clinicopathological parameters cannot yield satisfying performance to guide the individualized therapy. Integrating molecular biomarkers such as CIMP epigenotype seems to be promising in improving the predictive accuracy. Therefore, a prospective study with abundant case numbers of stage II patients is needed to evaluate the prognostic value of CIMP status in order to improve individualized therapeutic strategies.

## 5. Conclusions

To conclude, our data suggested that CIMP positive was an unfavorable independent prognostic factor in stage II/III sporadic colorectal cancer in Chinese population. Stage II/III patients with CIMP positive epigenotype may not benefit from 5-fluorouracil based adjuvant chemotherapy. Further studies are merited to confirm the potential role of CIMP status assessment as a high risk factor improving the stratifying of stage II/III colorectal cancer. If CIMP positive epigenotype was validated as a marker for chemoresistance of 5-fluorouracil and oxaliplatin based standard regimen, the benefit of these patients from irinotecan based chemotherapy should be evaluated.

## Figures and Tables

**Table 1:** Clinicopathological characteristics associated with CIMP status.

	CIMP−	CIMP+	*P* value
	(*n* = 38)	(*n* = 12)	
	No. (%)	No. (%)	
Age (yr)			0.270
Mean ± SD	53.5 ± 11.9	57.8 ± 11.2	
Sex			0.750
Male	17 (44.7)	6 (50.0)	
Female	21 (55.3)	6 (50.0)	
Site^a^			0.046
Proximal	5 (13.2)	5 (41.7)	
Distal	33 (86.8)	7 (58.3)	
Stage			0.022
II	17 (44.7)	1 (8.3)	
III	21 (55.3)	11 (91.7)	
Histology			0.059
Adenocarcinoma	32 (84.2)	7 (58.3)	
Mucinous adenocarcinoma	6 (15.8)	5 (41.7)	
Grade			0.007
Well/moderate	31 (81.6)	5 (41.7)	
Poor	7 (18.4)	7 (58.3)	
Lymphovascular invasion			0.121
Present	3 (8.1)	3 (25.0)	
Absent	34 (91.9)	9 (75.0)	
Perineural invasion			0.961
Present	3 (7.9)	1 (8.3)	
Absent	35 (92.1)	11 (91.7)	
CEA level			0.802
Normal	28 (80.0)	10 (83.3)	
Elevated	7 (20.0)	2 (16.7)	
Adjuvant chemotherapy			0.637
Not received	10 (26.3)	4 (33.3)	
Received	28 (73.7)	8 (66.7)	

SD: standard deviation, CIMP: CpG island methylator phenotype, CEA: carcinoembryonic antigen.

^a^Proximal location included the cecum, ascending colon, hepatic flexure of colon, and transverse colon while distal location included the splenic flexure of colon, descending colon, sigmoid colon, and rectum.

**Table 2:** Univariate and multivariate analysis of the prognostic effect of CIMP status and clinicopathological features in stage II/III cases for DFS.

	Univariate analysis			Multivariate analysis^b^		
	No.	5 yr DFS	*P* value	HR	95% CI	*P* value
Age (yr)			0.182			
≤55	30	69.7%				
>55	20	55.0%				
Sex			0.990			
Male	23	64.6%				
Female	27	63.0%				
Stage			0.049			0.242
II	18	83.3%		1	reference	
III	32	52.9%		3.075	(0.468–20.205)	
Site^a^			0.092			
Proximal	10	40.0%				
Distal	40	69.6%				
Histology			0.671			
Adenocarcinoma	39	66.7%				
Mucinous	11	53.0%				
Grade			0.681			
Well/moderate	36	66.7%				
Poor	14	56.3%				
CEA level			0.304			
Normal	38	56.3%				
Elevated	9	44.4%				
Adjuvant chemotherapy			0.849			
Not received	14	71.4%				
Received	36	61.0%				
CIMP epigenotype			0.014			0.019
Negative	38	73.7%		1	reference	
Positive	12	31.3%		2.935	(1.193–7.220)	

5-yr DFS: five-year disease-free survival, CIMP: CpG island methylator phenotype, CEA: carcinoembryonic antigen, HR: hazard ratio.

^a^Proximal location included the cecum, ascending colon, hepatic flexure of colon, and transverse colon while distal location included the splenic flexure of colon, descending colon, sigmoid colon, and rectum.

^b^Only factors which showed significant impact on DFS in the univariate analysis were included in the Cox regression analysis.

**Table 3:** Univariate analysis of the prognostic effect of CIMP status and clinicopathological features in stage II/III cases for OS.

	Univariate analysis^b^		
	No.	5-yr OS	*P* value
Age (yr)			0.078
≤55	30	74.2%	
>55	20	58.5%	
Sex			0.459
Male	23	60.5%	
Female	27	74.4%	
Stage			0.222
II	18	78.2%	
III	32	62.2%	
Site^a^			0.117
Proximal	10	50.0%	
Distal	40	82.0%	
Histology			0.559
Adenocarcinoma	39	64.3%	
Mucinous	11	81.8%	
Grade			0.820
Well/moderate	36	65.2%	
Poor	14	56.3%	
CEA level			0.255
Normal	38	71.4%	
Elevated	9	50.0%	
Adjuvant chemotherapy			0.083
Not received	14	35.7%	
Received	36	78.0%	
CIMP epigenotype			0.354
Negative	38	69.0%	
Positive	12	65.6%	

5-yr OS: five-year overall survival, CIMP: CpG island methylator phenotype, CEA: carcinoembryonic antigen, HR: hazard ratio.

^a^Proximal location included the cecum, ascending colon, hepatic flexure of colon, and transverse colon while distal location included the splenic flexure of colon, descending colon, sigmoid colon, and rectum.

^b^Only univariate analysis was performed since all factors considered were not significantly associated with OS.
