# Erratum to “Prognostic and Predictive Value of CpG Island Methylator Phenotype in Patients with Locally Advanced Nonmetastatic Sporadic Colorectal Cancer”

**DOI:** 10.1155/2014/418148

**Published:** 2014-03-26

**Authors:** Yuwei Wang, Yadong Long, Ye Xu, Zuqing Guan, Peng Lian, Junjie Peng, Sanjun Cai, Guoxiang Cai

**Affiliations:** ^1^Department of Colorectal Surgery, Fudan University Shanghai Cancer Center, 270 Dong An Road, Shanghai 200032, China; ^2^Department of Oncology, Shanghai Medical College, Fudan University, 270 Dong An Road, Shanghai 200032, China

The correct full authors' addresses should be as follows:

Department of Colorectal Surgery, Fudan University Shanghai Cancer Center; Department of Oncology, Shanghai Medical College, Fudan University, 270 Dong An Road, Shanghai 200032, China.

